# A Comparative Study Across Methods to Identify Adolescents with Syntactic Specific Language Impairment

**DOI:** 10.21315/mjms2022.29.6.13

**Published:** 2022-12-22

**Authors:** Hui Ying Jong, Abdul Rauf Rozaida, Jafri Malin Abdullah, Mohammed Faruque Reza, Garry Kuan

**Affiliations:** 1 School of Humanities, Universiti Sains Malaysia, Pulau Pinang, Malaysia; 2 Department of Neurosciences, School of Medical Sciences, Universiti Sains Malaysia, Kelantan, Malaysia; 3 Brain and Behaviour Cluster, School of Medical Sciences, Universiti Sains Malaysia, Kelantan, Malaysia; 4 Department of Neurosciences and Brain Behaviour Cluster, Hospital Universiti Sains Malaysia, Kelantan, Malaysia; 5 School of Medical Sciences, Universiti Sains Malaysia, Kelantan, Malaysia; 6 Exercise and Sports Sciences, School of Health Sciences, Universiti Sains Malaysia, Kelantan, Malaysia

**Keywords:** conventional method, software-assisted method, syntactic specific language impairment, typically developing, language assessments, E-Prime 2.0

## Abstract

**Background:**

Specific language impairment (SLI) is described as a heterogeneous deficit that causes difficulties in various aspects of language. We performed a comparative study of two methods of language assessment with the primary objective of determining the most effective approach for identifying adolescents with syntactic SLI and typical development (TD) in use.

**Methods:**

A software-assisted method using E-Prime 2.0 was used to create an experiment. The participants were Malay adolescents aged 13 years old–15 years old. The conventional method was compared with the software-assisted method to assess the participants’ comprehension and production performance. Data on reaction time (RT), scoring and no response (NR) were obtained from the adolescents.

**Results:**

Based on the two methods, the findings on the selection of participants for the SLI and TD groups was different. The two methods produced similar results in terms of the selection of TD group and most participants in the syntactic SLI group except for two participants who failed in the conventional method but passed the test in the software-assisted method.

**Conclusion:**

The descriptive evaluation of the findings suggested selecting software-assisted method as the alternative source because the provided information was detailed and this information enabled the researcher to identify the SLI group.

## Introduction

Specific language impairment (SLI) is characterised by a communication disorder that interferes with the development of language skills, such as word finding, phonology, morphology, syntax, semantics and pragmatics (1), whereas non-verbal cognition remains intact. Conventional clinical language tests, such as the Battery for Assessment of Syntactic Abilities (BAMBI) (2), are commonly used in several clinical and therapeutic settings, and they are efficient in detecting syntactic difficulties, predominantly for children with syntactic SLI (3–5), hearing impairment or deafness and hard-of-hearing (6–10), as well as individuals with agrammatism (11).

E-Prime (2004) is a programme designed to use a computer as an interface to facilitate the conception of any experiment between a subject and an experimenter. There are a few applications that the experimenter can run with E-Prime, including carrying out the experiment, collecting results, conducting some basic data analysis and exporting data. A considerable amount of literature has revealed that the application of E-Prime is useful for experimentation, notably for psychology studies and studies focusing on syntactic SLI, and researchers have widely investigated its probe accuracy and response time or reaction time (RT) for syntactic and lexical processing or even procedural learning (12–14). Furthermore, many attempts have been made by researchers to investigate real-time syntactic processing among participants syntactic SLI. Using E-Prime in conjunction with electroencephalography (EEG) (e.g. Net Station), a workstation has been developed that allows researchers to take advantage of the flexibility of E-Prime in the demanding environment of event-related potential (ERP) experiments (15–17) and in view of the fact that electrophysiological methods may reveal underlying immaturity or other abnormality of auditory processing even when behavioural thresholds look normal (15).

In an applied setting, we deal with individuals who have already been subjected to several assessments or who are even unmotivated to complete a long task. This phenomenon is unavoidable and it is likely to produce lapses in their attention on a task. As a consequence, there will be a reduction in the observed reliability and validity estimates. Therefore, a trade-off between the increased reliability and validity promoted by a lengthy task and the decreased reliability and validity related to the extended testing periods will emerge in an experiment. One way to reconcile this trade-off is to allow individuals a sense of control over administration (18) in the experiment by using E-Prime software; this is the primary advantage of using E-Prime software. A second advantage of using E-Prime is the randomisation function in this software. The selection and use of the randomise function in an experiment adds to its rigorousness (18–20). E-Prime contrasts with the conventional method, in which a researcher has to arrange the order of a trial manually, because a randomised trial can be utilised in the software. Audio output devices can also be used in the experiment to deliver audio stimuli. In the current study, the researcher recorded the voice of a Malay female volunteer, who read all the tested sentences and presented the sentences to the participants. In the conventional method, varying intonation or stress (changing intonation) during reading can completely change the meaning of a sentence. In other words, inconsistency in audio delivery can be crucial in an experiment.

As a third advantage, E-Prime software can provide feedback concerning the response accuracy on every trial (18, 19, 21). This allows participants to be informed about their performance. In contrast, the binary picture matching task in our study did not allow feedback on accuracy until the task was completed, which means that participants were informed of their accuracy (received feedback later) but they remained unaware of their performance during the task; this choice was made to encourage them to concentrate on their task rather than their performance in terms of whether they were producing optimal performance during the task. According to Voyer et al. (18), computer tasks enable the production of summary statistics for individual participants or group data; hence, they can eliminate scorer errors. For the conventional method, the paper-and-pencil format was required (22, 23) and the calculation of scoring had to be performed by the researcher manually. For the comparison between the manual calculation of scoring and RT, the calculation procedure of RT is definitely error prone and lengthy. Here, RT in E-Prime is the utmost additional source of information for comprehension response. In addition, allowing the researcher to retrieve information about the speed-accuracy trade-off, it also adds elements of interpretation, especially when the speed of comprehension is lower or higher than it is in the control group.

The advantages of E-Prime listed above suggest that examining participants directly on it has much potential for future use, especially the quantity and quality of data obtained, although the advantages of E-Prime do not necessarily imply that it has a high level of reliability and validity. The application of E-Prime software to examine or diagnose the comprehension of Wh- questions among Malay adolescents with syntactic SLI has not yet been implemented empirically in Malaysia. Thus, in the present study, we assessed the comprehension performance of Wh- questions using two methods—the conventional and software-assisted methods—among SLI adolescents and a control group (referred to as the typical development [TD] group). We intended to compare these two methods, primarily to observe the similarity of participant selection in the syntactic SLI and TD groups. Broadly, this research has the following aims: i) to identify adolescents with syntactic SLI and TD when using conventional methods and software-assisted methods and ii) to compare the effectiveness of conventional method and software-assisted method in selecting adolescents with syntactic SLI and TD.

## Methods

### Study Design

To identify adolescents with syntactic SLI and TD, this study used the conventional method and software-assisted method. The data were collected only once. Using the two methods, comprehension and production tests were observed and compared among participants.

### Sample Size Calculation

The required sample size was estimated using G*Power version 3.0.10. With a hypothesised effect size of 0.65, alpha of 0.05, power of 0.80, three groups and two measurement occasions, the estimated sample size was 27. After adding the estimated dropout rate of 10%, a sample of 30 adolescents was deemed sufficient to detect the magnitude of within-between differences that was expected. The total number of adolescents in the syntactic SLI group was 20 Malay adolescents (randomly assigned into groups with 10 adolescents with an intervention and 10 adolescents without intervention), while there were 10 adolescents in the TD group. However, in this study, we focused on comparing two methods for selecting the syntactic SLI and TD groups instead of the intervention. As a result, subtracting 10 from 20 adolescents in the syntactic SLI group, we obtained 10 adolescents in the syntactic SLI group in this study. This approach was chosen to obtain a similar number of adolescents between the syntactic SLI and TD groups in this study.

### Participant Selection and Screening

At the initial stage, 67 Malay adolescents aged between 13 years old and 15 years old were approached. All the participants attended regular classes in a national secondary school. There were three screening tests in this study, which were as follows: i) exclusion criteria; ii) non-verbal intellectual functioning and iii) clinical language tests. In the first screening test, the researcher aimed to exclude participants who met the exclusion criteria related to syntactic SLI (24)—namely, those with hearing impairments or recent episodes of otitis media, abnormalities of oral structure or problems in oral function, evidence of obvious neurological impairment or impaired neurological development and symptoms of impaired reciprocal social interaction or restriction of activities that are typical of autism or pervasive developmental disorders. The second screening test was performed to ensure that participants’ non-verbal level of intellectual functioning was age appropriate. Non-verbal intellectual functioning was indicated by participants’ scores on the Raven’s Matrices test, in which they performed within 1 standard deviation (SD) of the average for adolescents of their age. If all the criteria in the first and second screening tests were fulfilled, the participants proceeded to the third screening test. The purpose of the third screening test was to examine the participants’ syntactic abilities, which was done via a conventional clinical language test. Participants were randomly included in the syntactic SLI or TD group based on their performance on four syntactic comprehension and production tests. In total, 35 Malay adolescents fulfilled the selection criteria. Based on their performance, 20 were randomly assigned to the syntactic SLI and TD groups, with 10 in each group.

Next, we implemented the software-assisted method using E-Prime software. This was a crucial stage whereby we compared the results between conventional clinical language tests and software-assisted methods.

### Clinical Language Tests

#### Conventional Method

The participants were diagnosed with syntactic SLI using the four following syntactic language tests: two comprehension tests of relative clauses and two production tests of relative clause production (4, 26). Participants were included in the syntactic SLI group if they failed in at least two out of the four tests. Failure in a test was defined as a performance that was significantly poorer than the mean score of TD children and was tested using the receiver operating characteristic (ROC) for the comparison of a single subject to a group, with an alpha level of 0.05.

### Comprehension tests I and II

To identify comprehension impairment among adolescents with syntactic SLI, participants were assessed using a binary sentence-picture matching task (BAMBI ZTI) and a task of comprehension questions (BAMBI ZIKA MEGUVANA) (4, 5). In BAMBI ZTI, each participant was verbally presented with 40 subject relative (SR) clauses, object relative (OR) clauses and subject-verb-object (SVO) sentences; all the sentences were read by a native speaker of the Malay language. Participants listened to a semantically reversible sentence and saw two pictures on the same page. They were asked to point to the picture that correctly described the sentence, selecting between two pictures presented, one was at the top and the other was at the bottom; the roles matched the sentence in one picture, whereas they were reversed in the other picture. All verbs were agentive transitives.

In BAMBI MEGUVANA, participants listened to 90 subject questions, object questions and SVO sentences. They answered questions that required the comprehension of the thematic roles in the relative clauses.

### Production tests I and II

The production of relative clauses was assessed using two elicitation tasks—a preference task and a picture description task. For the preference task, the preference question aimed to evoke the production of six SR clauses and six OR clauses. In the elicitation task, for the purpose of eliciting both relative clauses, we used a description of picture pairs, such as the picture pair in Figure 2; the first picture described one figure performing an action on the other, while in the second picture, the roles were reversed. The researcher used simple sentences to describe the two pictures, and then participants were asked about one of the figures and its role in each of the pictures; the target response was either an SR or OR clause. Ten SR clauses and 10 OR clauses were included in this picture pair description task.

#### Software-Assisted Method

The comprehension of Wh- questions was tested using a binary picture matching task (Figure 2) and this task was presented using a psychology software tool—namely, E-Prime version 2.0. Building a clinical language test is a process consisting of several distinct stages; here, each stage had certain deliverables and was bound by a specific time frame (Figure 3). Depending on the clinical language test, certain stages gained additional weight in the overall effort to implement the test. Several aspects associated with culture (or context) had to be considered when building the language test—for instance, a picture pair and all verbs (action that referred to the picture pair) used in the binary picture matching task.

The research stage was the stage in which requirements to build a clinical language test had to be formulated properly, for instance, the use of desktop or laptop, the picture pair presented one above the other with the roles were reversed in second picture and having a native speaker of Malay read the sentences for the purpose of auditory presentation. Furthermore, a set of goals had to be defined—for instance, goals were set that the picture pairs and verbs used had to be clear and understandable and the participant had to have sufficient time to respond. Then, the planning stage was the stage in which all the elements were set to develop in the clinical language test. Planning starts with defining the overall flow of the clinical language test’s application. The next step was to break down the flow into smaller, easier to manage sub-assemblies—for instance, fixation, blank pages, sentences, audio recordings and picture pairs. The third stage was design. This was the stage in which the layout of the application was created. The graphic design stage was important because it would display to the participant a preview of the application before it was actually built. At this stage, the researcher came up with new requirements that had to be submitted to research and planning. For example, the use of colour in the picture pairs needed to be consistent.

The fourth stage was prototyping. At this stage, specifications needed to be better defined; this stage also entailed building a demo version of a clinical language test that included the critical functionality. Fifth, participants’ feedback was received after the prototype was completed. Sixth, the development stage was the stage in which the clinical language test was actually built. This stage started with setting up the development and testing environments. In this study, these environments were set up in secondary schools. Both environments were synchronised using the same protocol. The next stage was testing. This was a stage in which the design errors were identified and fixed. Following this, the researcher conducted the set-up stage. The application of the clinical language test was installed and the back-up procedures (two or more desktops installed with E-Prime software) were defined and tested. Once the application of the clinical language test was installed, it went through another full testing cycle. Finally, the maintenance stage was responsible for ensuring that the application of the clinical language test was running within planned parameters and that the traffic data would provide valuable input (i.e. scoring, RT, and no response [NR]) on potential issues that could affect the application’s performance. The output of the prototyping methodology is shown in Figure 4.

Participants were presented with a total of 80 Malay questions each, 20 of each type (20 ‘who’ *siapa* subject questions [SS], 20 ‘which’ *yang mana* subject questions [SM], 20 ‘who’ *siapa* object questions [OS] and 20 ‘which’ *mana* object questions [OM]). The participants were tested individually in a quiet room. The questions were randomly ordered and no more than two questions of the same type appeared consecutively. The participant saw the picture pairs four times; each picture pair appeared with each of the four question types, once in each session. The correct picture in each pair was randomised in each session; half the sentences matched the upper picture, and half matched the lower picture, pointing to the same picture position no more than three consecutive times. All verbs were agentive transitives and all the questions were semantically reversible so that comprehension of the meaning of the words alone could not determine the meaning of the sentence (i.e. we did not use irreversible sentences, such as ‘Which girl is eating a sweet?’, only reversible sentences, such as ‘Which girl is kissing the mother?’). For this task, the experimental questions were followed by a fixation symbol ‘+’ (500 ms), a first blank (500 ms), then a sequential presentation (5,000 ms) of one of the four question types, auditory presentation of each question (7,000 ms) and a second blank (500 ms). Finally, a binary picture matching was displayed (8,000 ms) on the upper and the lower sections of the laptop screen, and participants were requested to press either ‘1’ (upper picture) or ‘2’ (lower picture; see Figure 2). The Wh- question and auditory presentation were in one occurrence. The auditory presentations were digitally recorded by a female native speaker of Malay. Measures of scoring, RT and NR were obtained from E-Prime.

### Statistical Analysis

**Figure 1:**
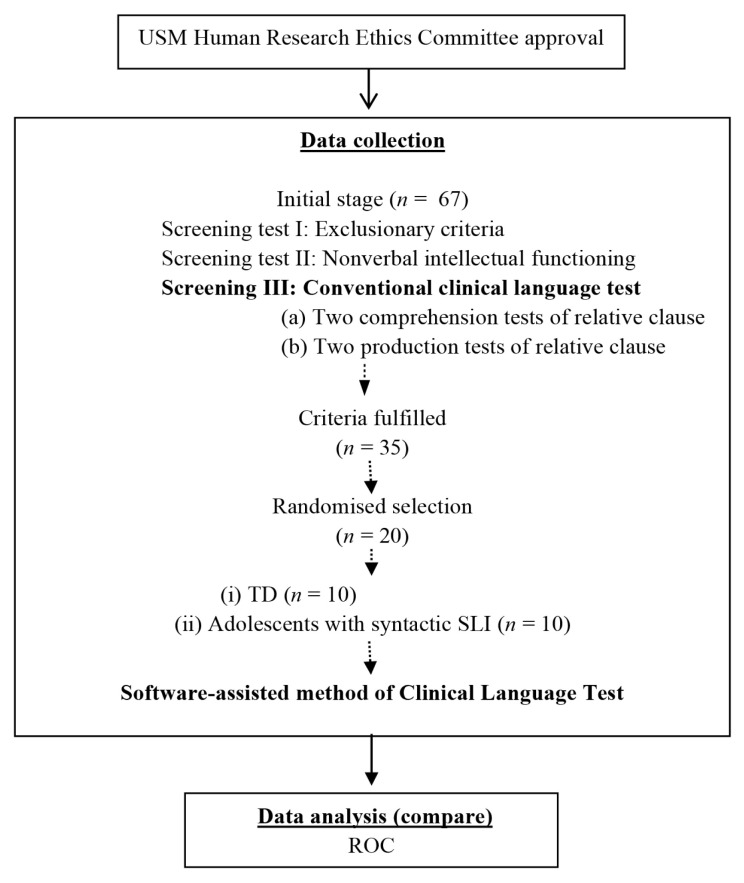
Flowchart of the study design

**Figure 2:**
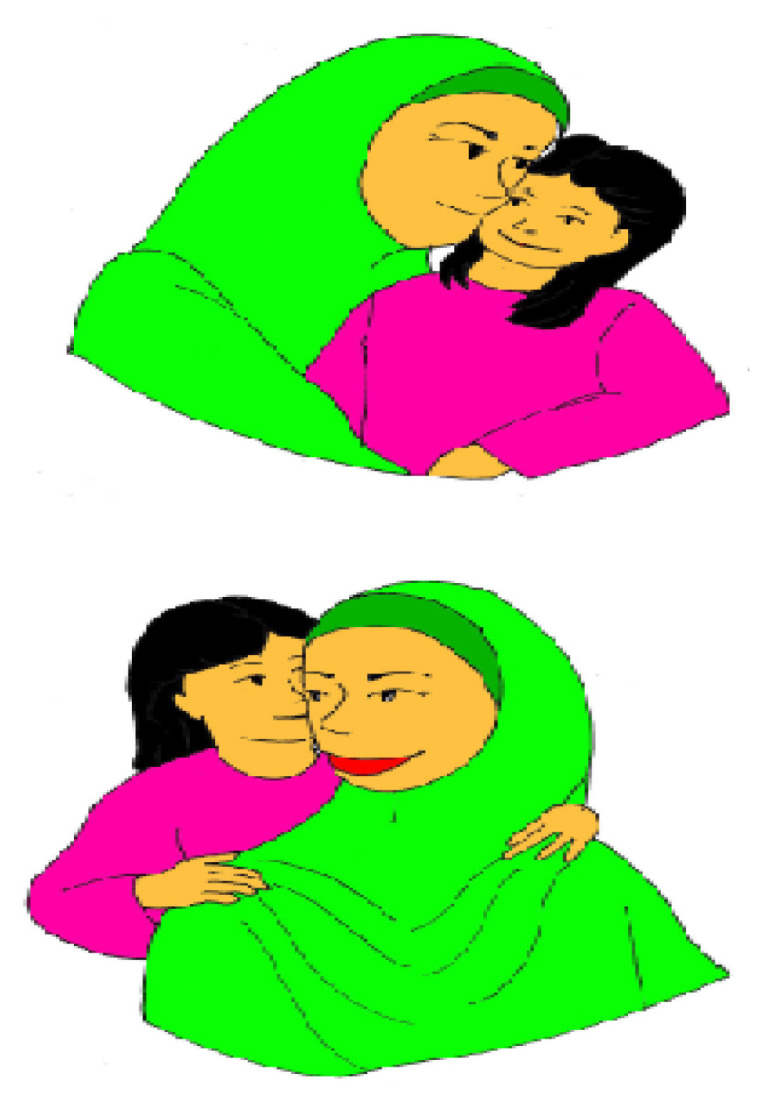
Example of a binary picture matching task

**Figure 3:**
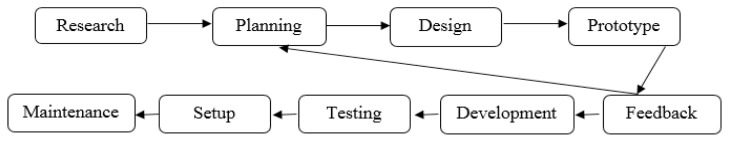
Prototyping methodology

**Figure 4:**
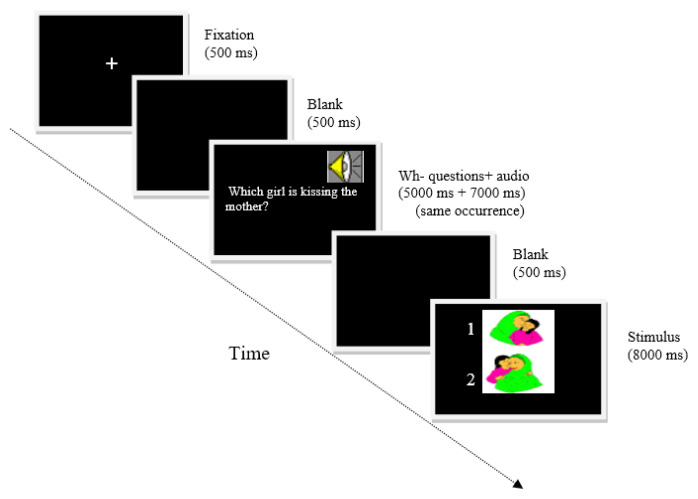
Block diagram of algorithm showing the binary picture matching task

Data analysis was conducted using the Statistical Package for the Social Sciences (IBM SPSS Statistics version 24.0). In this study, failure in a test was defined as a performance that was significantly poorer than the mean score of the TD group and was tested using ROC for the comparison of a single subject to a group. In other words, ROC was used to distinguish between TD adolescents and adolescents with syntactic SLI. ROC curves are a useful way to interpret sensitivity and specificity levels and to determine related cut scores, as well as a generalisation of the set of potential combinations of sensitivity and specificity possible for predictors (25). An overall indication of the diagnostic accuracy of the ROC curve is the area under the curve (AUC). To identify the separability between the distributions of scores for the positive and negative populations, the AUC is the perfect measurement. The AUC summarises the results over all possible choices, instead of requiring one to choose a threshold value. To compute a ROC curve in SPSS, the screening measure of conventional and software-assisted tests used a standard score, and the outcome measures were recorded into a dichotomous variable of Not at-risk = 0 and At-risk = 1 (Table 1). A flow chart of the study design is shown in Figure 1.

### Ethical Considerations

The study received approval from the Universiti Sains Malaysia (USM) Human Research Ethics Committee. Prior to data collection, the adolescents received an explanation of the research. All adolescents were sent a written consent form along with a separate participant information sheet. They were asked to read the consent form and their signatures were taken before the start of the experiment. In our study, consent was also obtained from participants’ parents or guardians.

## Results

Table 1 compares the results obtained from the preliminary analysis of comprehension and production tests in conventional and software-assisted methods. As can be seen from Table 1, for relative clauses, there are two types of comprehension tests and two types of production tests in the conventional method—BAMBI ZTI, BAMBI ZIKA MEGUVANA, BAMBI ZIBUV and BAMBI ADIF. To observe the participants’ language performance in the conventional method, scorings were used. To observe comprehension of Wh- questions in the software-assisted method, scoring, RT and NR were used. Failure in a test was defined as a performance that was significantly poorer than the mean score for TD children and was tested using ROC for the comparison of a single subject to a group, with an alpha level of 0.05. To compute a ROC curve in SPSS, the screening measure of conventional and software-assisted tests used a standard score, and the outcome measures were recorded into a dichotomous variable of Not at-risk = 0 and At-risk = 1. The results of the conventional and software-assisted methods are summarised in Table 2. From the table, it can be seen that by far the poorest performance was found for participants B1 to B10.

In the conventional method, of the 20 adolescents who took part in the BAMBI ZTI test, nine and five participants in SLI group performed significantly poorer compared with the TD group on SVO and SR, respectively. The correlation between the BAMBI ZTI test and BAMBI ZIKA MEGUVANA is noteworthy because the data comparison demonstrates that half the adolescents performed more poorly than the other participants on OR in both comprehension tests and it is interesting to note that they were the same adolescents (participants B1, B2, B3, B4, B5, B6, B7, B8, B9 and B10). In the comparison of first production performance (BAMBI ZIBUV) of each adolescent in each question type to the TD group, only five adolescents (participants B1, B2, B5, B6 and B9) performed significantly more poorly than the TD group on SR. Unexpectedly, for a small number of participants (only three) on the second production test (BAMBI ADIF; participants B5, B6 and B8), it was also found that they performed poorly on SR. The marked observation to emerge from the data comparisons was that the 10 adolescents, as mentioned in terms of the comprehension tests, also failed on OR in these two types of production tests.

Conversely, the scoring of the Wh- questions in the software-assisted method indicated that only five adolescents (B1, B2, B3, B9 and B10) from the syntactic SLI group performed more poorly compared with the TD group on the OM Wh- question. (OM has a similar structure to OR). The results showed that they performed poorly on OM questions, as well as on the SS, OS and SM Wh- questions. For the RT of Wh- questions, our experimental results were consistent for BAMBI ZTI and BAMBI ZIKA MEGUVANA, with 10 adolescents from the syntactic SLI group performing significantly more poorly (B1, B2, B3, B4, B5, B6, B7, B8, B9 and B10) than those in the TD group. Adolescents from the syntactic SLI group took longer to interpret the four types of Wh- questions. For NR, the result of SM in the syntactic SLI group was like that in the TD group; only one adolescent from the syntactic SLI group performed more poorly than those in the TD group; for SS and OS, five and six adolescents, respectively, from the syntactic SLI group gave similar responses to those in the TD group. They performed well on these two types of Wh- questions.

In contrast to the SM, 70% of the adolescents from the syntactic SLI group performed significantly poorly on the OM. The participants were diagnosed with syntactic SLI if they failed on at least two measurements in this software-assisted method in terms of scoring, RT or NR. Thus, a considerable syntactic impairment in the comprehension of Wh- questions was established at the group level, as well as for each individual with syntactic SLI. The 10 adolescents with syntactic SLI who participated in the four conventional clinical language tests were compared with tests using the software-assisted method. As a result, eight adolescents with syntactic SLI who failed in the conventional clinical language tests also failed in the clinical language test using the software-assisted method. Of the eight adolescents with syntactic SLI, four failed in all three measurements (B1, B3, B9 and B10), whereas the others (B2, B4, B5, B6, B7 and B8) failed in two measurements in the software-assisted method. Unexpectedly, adolescents B5 and B6, who failed on the conventional clinical tests, only failed in one measurement using the software-assisted method. The summary of each test in Table 1 is shown in Table 2.

## Discussion

Prior studies have noted the importance of the software- or computer-assisted method in the field of language assessment (26–29). However, in reviewing the literature, no data was found comparing the conventional and software-assisted methods in the selection of normal or typically developing and impairment groups with the purpose of observing the similarity in the selection of participants. This study set out to compare the selection of participants into the SLI and TD groups between the conventional method and the software-assisted method. Given that the difference in selection of participants into syntactic SLI and TD groups may be of great importance, particularly in applied settings, it is worthwhile to explore the difference between the two methods of the tasks without regard for the outcome of comparisons. From this perspective, one is compelled to conclude that the software-assisted method involving E-Prime is likely more appropriate than the conventional method for the selection of participants into syntactic SLI and TD groups. Specifically, the comprehension task in the software-assisted method clearly specified that participants B5 and B6 did not have syntactic SLI as they only failed in one test.

Although the results of the conventional task were able to show a clear difference between syntactic the SLI and TD groups on the comprehension and production tests, our findings suggest that the software-assisted method may be a better assessment method simply because, unlike the conventional approach, it produced detailed information. In addition, since most researchers are likely to use only one testing session (either with the conventional or software-assisted method), and since the software-assisted method of using E-Prime enables the researcher to obtain exactly the same information and more compared with the conventional method instead of scoring the results, the findings of this study favour the software-assisted method.

At this point, one may wonder why similar questions were not used for both the conventional and software-assisted methods in the present study. For example, the researcher could have used a similar language test of relative clauses in both the conventional method and the software-assisted method. This would have yielded more directly comparable results and minimised the influence of different materials on sentence testing. However, the test-retest administration of the two methods for the clinical language tests would certainly result in fatigue and boredom. It is quite likely that these factors would increasingly affect the results as the testing progressed, possibly resulting in lower concentration and lower performance in RTs or accuracy (30, 31). In addition, the effects of the same material used in testing could also cause improvement stemming from repetition because participants’ would be given more chance to practice and become familiar with the task (32). From the reliability perspective, a very high test reliability will be obtained if the items are too easy because the participants will always get the items right. The same effect will also be seen on test-retest reliability for difficult items because participants will always fail to answer them (33). As a consequence, another set of syntactic material was designed for the comprehension test. Instead of using relative clauses as in the conventional method, we used Wh- questions in the software-assisted method. Relative clauses and Wh- questions possess the same syntactic structure, derived from Wh- movement; thus, comprehension of these structures involves similar abilities. From this perspective, it appears that although the syntactic material used here was different (but had the same syntactic structure), it likely minimised the influence of external factors, such as participants’ fatigue or attrition.

The results of the present study underline at least two points regarding the software-assisted clinical language task considered here that require further discussion. First, the software-assisted method appears to provide a better assessment of comprehension. Second, the results of the comprehension test using the software-assisted method were clearer than those of the conventional method. The main effect of procedure on correct responses, reflecting an advantage for the software-assisted method, is one finding that clearly suggested possible differences between these two methods. The finding in the software-assisted method suggests that the correct responses, namely scoring of Wh- questions among participants B5 and B6, were like those in the TD group. If and only if the participant is unable to give the response to the Wh- questions within the fixed RT will the scoring of correct responses be affected because E-Prime software automatically computes or counts this as ‘0’. In other words, it is a wrong response. Measurement of NR will also be indicated as ‘0’ in E-Prime. In some way, this condition will reflect the disadvantage of the software-assisted method owing to its lack of flexibility. As researchers, we can assume that the participant was not concentrating on the task or was even affected by other external or internal factors. In contrast with the conventional method, in the software-assisted method, it was obligatory for all participants to answer and complete the task. As a consequence, the researcher will never obtain the information on NR, which are important data mainly relating to participants who are unable to comprehend the syntactic structure and fail to give a response.

These are important findings. Therefore, it can be stated that the comparison between conventional and software-assisted methods provides some insight that software-assisted methods may be crucial in language tests or assessments. The findings may help us to design, develop or transform the conventional method to a software-assisted approach. In future investigations, it may be possible to use software-assisted methods in different settings, such as hospitals, speech pathology clinics or even educational settings. Further studies are also needed to examine the effects of the software-assisted method when it is combined with other educational technology tools. Although the current study was based on a small sample of participants, the results are still encouraging. To better understand the effectiveness of implementing the software-assisted method in clinical or educational settings, the method should be validated by including a larger sample size in future studies.

## Conclusion

The above discussion shows that the software-assisted method of E-Prime represented an improved version subsequent to the pilot testing of E-Prime. For example, it seems plausible to suggest that the appropriate RT for each trial, removal of feedback and having a short break between the sessions might affect the performance positively, considering that a lack of concern for these issues will cause the participants to be unable to perform better or more accurately in the task. Furthermore, the extended response duration provided in the conventional method allowed participants sufficient time to change their responses. For the software-assisted method, this should be made possible in the test as well.

## Figures and Tables

**Table 1:** Comparison between conventional and software-assisted methods of clinical language testing

Subject	Conventional method										Software-assisted method											

	BAMBI ZTI			BAMBI ZIKA MEGUVANA			BAMBI ZIBUV		BAMBI ADIF		Comprehension of Wh- questions											

	Scoring			Scoring			Scoring		Scoring		Scoring				RT				NR			

	SVO	SR	OR	SVO	SR	OR	SR	OR	SR	OR	SS	OS	SM	OM	SS	OS	SM	OM	SS	OS	SM	OM
A1	0	0	0	0	0	0	0	1	0	0	1	0	0	0	0	0	0	0	0	0	0	0
A2	0	0	0	0	0	0	0	0	0	0	0	0	0	0	0	0	0	0	0	0	0	0
A3	0	0	0	0	0	0	0	0	0	0	0	0	1	0	0	0	0	0	0	0	0	0
A4	0	0	0	0	0	0	0	0	0	0	0	0	1	0	0	0	0	0	0	0	0	0
A5	0	0	0	0	0	0	0	0	0	0	0	0	0	0	0	0	0	0	0	0	0	0
A6	0	0	0	0	0	0	0	0	0	0	0	0	0	0	0	0	0	0	0	0	0	0
A7	0	0	0	0	0	0	0	0	0	0	0	0	0	0	0	0	0	0	0	0	0	0
A8	0	0	0	0	0	0	0	0	0	0	1	0	0	0	0	0	0	0	0	0	0	0
A9	0	0	0	0	0	0	0	0	0	0	0	0	0	0	0	0	0	0	0	0	0	0
A10	0	0	0	0	0	0	0	0	0	0	0	0	0	0	0	0	0	0	0	0	0	0
B1	1	1	1	0	0	1	1	1	0	1	1	1	1	1	1	1	1	1	1	0	1	1
B2	1	1	1	0	1	1	1	1	0	1	1	1	1	1	1	1	1	1	1	1	0	0
B3	1	0	1	0	0	1	0	1	0	1	1	1	1	1	1	1	1	1	0	0	0	1
B4	1	1	1	1	1	1	0	1	0	1	0	1	0	0	1	1	1	1	0	1	0	1
B5	1	0	1	0	0	1	1	1	1	1	0	0	0	0	1	1	1	1	0	0	0	0
B6	0	0	1	0	0	1	1	1	1	1	1	1	0	0	1	1	1	1	0	0	0	0
B7	1	1	1	0	0	1	0	1	0	1	1	1	0	0	1	1	1	1	1	0	0	1
B8	1	0	1	0	0	1	0	1	1	1	1	1	0	0	1	1	1	1	1	1	0	1
B9	1	1	1	0	0	1	1	1	0	1	0	0	1	1	1	1	1	1	0	0	0	1
B10	1	0	1	0	0	1	0	1	0	1	1	0	1	1	1	1	1	1	0	0	0	1

Notes: SVO = subject-verb-object; SR = subject relative; OR = object relative; SS = subject *Siapa*; OS = object *Siapa*; SM = subject *Mana*; OM = object *Mana*

**Table 2:** Summary of each test reported in Table 1

Methods	Comprehension/Production		%	Type of Sentence	TD	SLI
Conventional method	Comprehension	BAMBI ZTI	Scoring	SVO	100	10
				SR	100	40
				OR	100	0
		BAMBI ZIKA MEGUVANA	Scoring	SVO	100	90
				SR	100	70
				OR	100	0
	Production	BAMBI ZIBUV	Scoring	SR	100	50
				OR	100	0
		BAMBI ADIF	Scoring	SR	100	70
				OR	100	0
Software-assisted method	Comprehension		Scoring	SS	80	30
				OS	100	30
				SM	90	50
				OM	100	50
			Response Time	SS	100	0
				OS	100	0
				SM	100	0
				OM	100	0
			No Response	SS	100	60
				OS	100	70
				SM	100	90
				OM	100	30

Notes: TD = typically development; SLI = specific language impairment; SVO = subject-verb-object; SR = subject relative; OR = object relative; SS = subject *Siapa*; OS = object *Siapa*; SM = subject *Mana*; OM = object *Mana*
